# Comparing client and staff reports on tobacco-related knowledge, attitudes, beliefs and services provided in substance use treatment

**DOI:** 10.18332/tid/160974

**Published:** 2023-03-24

**Authors:** Cristina Martínez, Nadra Lisha, Caravella McCuistian, Elana Straus, Kevin Delucchi, Joseph Guydish

**Affiliations:** 1 Tobacco Control Unit, Cancer Control and Prevention Program, Institut Català d’Oncologia-ICO, Barcelona, Spain; 2 Cancer Control and Prevention Group, Institut d’Investigació Biomèdica de Bellvitge-IDIBELL, Barcelona, Spain; 3 Department of Public Health, Mental Health, and Maternal and Child Health Nursing, School of Medicine and Health Sciences, Universitat de Barcelona, Barcelona, Spain; 4 Institute for Health Policy Studies, University of California San Francisco, San Francisco, United States; 5 Centro de Investigación Biomédica en Red de Enfermedades Respiratorias (CIBERES), Madrid, Spain; 6 Department of Medicine, Division of General Internal Medicine, University of California San Francisco, San Francisco, United States; 7 Center for Tobacco Control Research and Education, University of California San Francisco, San Francisco, United States; 8 Department of Psychiatry and Behavioral Sciences, University of California San Francisco, San Francisco, United States

**Keywords:** substance use, smoking, tobacco policy, tobacco-related

## Abstract

**INTRODUCTION:**

Smoking is highly prevalent in substance use disorder (SUD) programs, but few studies have explored the tobacco-related attitudes of staff and clients in the same program. The aim of this study was to compare staff and client reports on 10 tobacco-related items and associate them with tobacco measures implemented in the programs.

**METHODS:**

A cross-sectional survey was conducted in 18 residential SUD programs from 2019 to 2020. Overall, 534 clients and 183 clinical staff self-reported their tobacco use, knowledge, attitudes, beliefs, and practices/services regarding smoking cessation. Ten comparable items were asked of both clients and staff. Differences in their responses were tested using bivariate analyses. We examine the association between selected tobacco-related items on making a quit attempt and planning to quit in the next 30 days.

**RESULTS:**

In all, 63.7% of clients were current cigarette users versus 22.9% of staff. About half of clinicians (49.4%) said they had the skills to help patients quit smoking, while only 34.0% of clients thought their clinicians had these skills (p=0.003). About 28.4% of staff reported encouraging their patients to use nicotine replacement treatment (NRT), and 23.4% of patients said they had been encouraged to use these products. Client reports of planning a quit attempt were positively correlated with whether both staff and clients reported that the use of NRT was encouraged (clients: r=0.645 p=0.004; staff: r=0.524 p=0.025).

**CONCLUSIONS:**

A low level of tobacco-related services was provided by staff and received by clients. In programs where smokers were encouraged to use NRT, a higher percentage of smokers planned a quit attempt. Tobacco-related training among staff, and communication about tobacco use with clients, should be improved to make tobacco services more visible and accessible in SUD treatment.

## INTRODUCTION

Worldwide, tobacco use among persons with substance use disorders (SUD) is between two to four times higher compared to the general population^1^. In the US, changes in the prevalence of self-reported cigarette smoking have been observed among this population in recent years, with a significant decline from 46.5% in 2006 to 35.8% in 2019^2^. Nonetheless, half of the smokers with SUD problems who received treatment services will die from tobacco-related diseases^3^.

In contrast to the belief that quitting tobacco may negatively affect abstinence from other substances, studies suggest that providing smoking cessation during SUD treatment improves abstinence from other drugs^4^ and those who were nicotine abstinent for one year have better long-term outcomes including drug abstinence and remission^5^. In addition, continued smoking was associated with greater odds of SUD relapse^6^.

As recommended by the Substance Abuse and Mental Health Service Administration (SAMHSA), receiving a smoking cessation intervention based on counseling and the provision of tobacco-dependence treatment during SUD treatment could increase the motivation to quit in a favorable and healthy environment, since quitting a primary drug could represent an opportunity to receive smoking cessation services^7^. According to one review, admission to a smoke-free psychiatric service reduced daily cigarette consumption post-discharge and increased both patient motivation to quit and quit attempts^8^. For these reasons, providing tobacco cessation treatment is recommended as the standard of care in SUD treatment programs^9^.

While one-third of US SUD treatment programs had smoking bans on their property^10^, residential SUD treatment programs are less likely to have tobacco-free grounds^11^. In addition, clinicians’ attitudes and behaviors towards smoking cessation programs appear to be influenced by the number of services provided and the extent of the tobacco control policies applied in their organizations^12,13^.

When measuring smoking cessation services, performance measures have been based either on provider documentation in the medical record, decision support, billing codes^14,15^, or surveys conducted among health professionals or clients^16^. However, patient and client surveys conducted at the point of service are considered optimal for measuring provider delivery of smoking cessation services^17^. A frequent limitation of these studies is that they report the view of one of those concerned (either professionals or clients). Only a few studies contrasted smokers’ and clinicians’ tobacco-related knowledge, attitudes, beliefs, and services received during substance use treatment^18,19^. Olsen et al.^19^, working with outpatient methadone treatment programs, found that counselors reported providing smoking cessation services to their clients more often that clients reported receiving such services. Cookson et al.^18^ conducted a similar analysis in inpatient addiction treatment clinics and found similar results.

No previous study has compared clinician and client reports from residential SUD treatment programs simultaneously and within the same program. Because clients live in the program center, the impact of tobacco-related policies may be greater than similar policies implemented in outpatient programs. The collection of both staff and client survey data in the same programs and at the same time point enables an ecological view of how patient behaviors may be affected by providers’ perceptions in the context of an organization.

The present study reports on 18 residential SUD treatment programs where both staff and client surveys included the same 10 items reflecting tobacco-related knowledge, attitudes, beliefs, and services/practices. We assess responses to these items among currently smoking clients and among clinical staff treating those clients. We also studied the association between the selected items and the outcome of whether clients made a quit attempt or planned to quit in the next 30 days.

## METHODS

### Program recruitment

In 2019, baseline data were collected from 18 publicly-funded residential SUD treatment programs in California. The programs were in 11 of California’s 58 counties and spanned over 500 miles from northern to southern California. Each program treated clients with substance use disorder, though some programs also provided treatment for mental health disorders, or for some other reason (e.g. preventive services for parolees re-entering the community). These programs were recruited to participate in one of three larger following studies. The first study, representing 7 of the programs, included programs that had applied to participate in a state-funded, tobacco policy-change intervention^20^. The second study included 7 programs that indicated an interest in implementing tobacco-free grounds in the course of a state-wide phone survey^21^. The third study included 4 residential SUD programs in San Francisco engaged in a community-based project to improve tobacco cessation services. Details of program recruitment are reported elsewhere^22^.

### Participants

Participants included staff and clients at each of the 18 programs. All paid staff (full-time and part-time) were eligible to participate. At each program, program directors provided email addresses for eligible staff, and each staff member was invited by email to complete an online survey. Reminders to complete the survey were sent to staff once a week for three weeks. After that time, research staff contacted program directors to discuss additional strategies to increase response rates. Responses were confidential and staff received a $25 gift card for completing the survey. In this report we have included only clinical staff, defined as a healthcare professional responsible for providing either care, counseling or therapeutical interventions addressed directly to clients. We included only clinical staff because these are the ones that could provide tobacco-related information and clinical services to clients.

Eligible clients were all those enrolled in the program at the time of data collection. The number of clients enrolled in the program, used to calculate response rates, was also reported by the program director. Client survey data were collected during in-person site visits to each program. Research staff explained the purpose of the study and reviewed the study consent sheet with clients. Consenting clients self-administered the survey on an iPad, and research staff were present to answer questions. Survey responses were anonymous, and clients received a $20 gift card for completing the survey. Although all clients were invited to participate in the survey, this report includes only those who self-reported as current smokers at the time of admission to the program. Apart from tobacco screening, only current smokers would have received tobacco-related services in the program.

### Measures

*Demographic information*

For both staff and clients, demographic characteristics included age, gender, race/ethnicity, and education level.

*Tobacco use*

Current tobacco use for both staff and clients was defined as having used any type of tobacco product (cigarettes, e-cigarettes, cigars, cigarillos, smokeless tobacco) in the past 30 days^23^. Smoking was categorized as current smokers (daily or occasional smokers of cigarettes in the past 30 days), former smokers (a smoker who had quit smoking), and never smokers (a person who had never smoked, or who has smoked less than 100 cigarettes in his or her lifetime)^24^. In the case of clients, to accurately measure the association between cigarette use and smoking cessation services provided in the SUD programs, we considered smokers only those who reported having used cigarettes in the past 30 days either exclusively or concurrently with other tobacco products (both combustible and non-combustible products that contain tobacco such as e-cigarettes, cigars, cigarillos).

Staff and clients who self-reported current cigarette smoking were asked about cigarettes smoked per day (CPD). Readiness to quit smoking was assessed with the question: ‘Are you seriously thinking of quitting smoking?’. Response codes were: ‘within the next 30 days’ or ‘next 6 months’, or ‘not thinking of quitting smoking’^25^.

*Smoking knowledge, attitudes, and practices/services*

Staff and client surveys included two scales specific to smoking-related issues in addiction treatment settings. Staff filled in the Smoking Knowledge, Attitudes, and Practices Scale (S-KAP)^26^. Whereas clients filled in the Smoking Knowledge, Attitudes, and Services Scale (S-KAS)^27^. From these two scales, we selected 10 items, with similar wording and response codes, which asked tobacco-related knowledge, about attitudes in the program toward quitting smoking, and about whether cessation services were available. Two of the ten items, asking whether the hazards of smoking were clear and whether counseling for smoking cessation was part of the program’s mission, were identical in the staff and client surveys. All other items had similar but not the same wording for both staff and clients. For example, staff were asked whether ‘my clients want to quit’, while clients were asked whether ‘clients that smoke in this program want to quit’. Other items asked whether counselors had the skills to help clients quit smoking, whether smoking was a personal decision and not a matter for counseling, whether clients were concerned about quitting smoking, and whether counseling by a clinician motivates clients to quit.

The remaining three items asked about tobacco related practices used by staff and received by clients. These included whether clients were asked about their smoking status, how often clients were encouraged to use nicotine replacement therapy (NRT), and how frequently clients were referred to a state quitline. Most items used the same response codes for both clients and staff, with responses on a 5-point Likert scale (strongly agree, agree, unsure, disagree, or strongly disagree). One item used a Likert response code from never to always for both clients and staff. Two items used a Likert scale for staff (how frequently did you ask clients whether they smoke, how frequently did you refer clients to a state quitline), while clients were asked if their counselor ever asked about smoking or referred them to a quitline (yes/no). The 10 selected items, with exact wording and response codes for clients and staff, are given in Table 2.

**Table 1:** Demographic comparison between clients and staff of the 18 clinics

*Characteristics*	*All clients (N=535) n (%)*	*Smoking clients (N=341) n (%)*	*Clinical staff (N=183) n (%)*
**Age** (years), mean ± SD	39.12 ± 11.66	37.57 ± 11.30	45.37 ± 12.21
**Gender**			
Male	400 (75.0)	248 (73.2)	63 (34.4)
Female	123 (23.1)	86 (25.4)	117 (63.9)
Other	10 (1.9)	5 (1.5)	3 (1.6)
**Race/ethnicity**			
Hispanic/Latino	207 (38.4)	120 (35.2)	47 (26.0)
Black or African American	102 (19.1)	59 (17.3)	38 (21.0)
White or Caucasian	169 (31.6)	126 (36.9)	73 (40.3)
Other/multiple	57 (10.6)	36 (10.6)	23 (12.7)
**Education level**			
Less than high school/GED	143 (26.7)	97 (28.4)	2 (1.1)
High school diploma or GED equivalent	194 (36.3)	127 (37.2)	88 (49.7)
More than high school/GED	198 (37.0)	117 (34.3)	87 (49.1)
**Cigarette users**			
Current	341 (63.7)	341 (100)	42 (22.9)
Former	131 (24.5)	-	92 (50.3)
Never	63 (11.8)	-	49 (26.8)
**Past month use of tobacco products**			
Cigarettes	341 (63.7)	160 (37.1)	50 (21.2)
E-cigarettes	121 (23.1)	110 (33.0)	38 (20.8)
Smokeless tobacco	72 (14.0)	61 (18.5)	7 (3.8)
Little filtered cigars or cigars	111 (21.7)	101 (31.0)	9 (4.9)
User of one or more products	363 (67.8)	341 (100)	114 (62.3)
**Cigarettes per day**		9.9 (7.6)	9.5 (6.5)
**Are you seriously thinking of quitting smoking?**			
Yes, next 30 days		111 (32.6)	17 (40.5)
Yes, within the next 6 months		108 (31.8)	18 (42.9)
No, not thinking of quitting smoking within the next 6 months		121 (35.6)	7 (16.7)
**Did you make a quit attempt in program? (Yes)**		112 (33.0)	

**Table 2:** Staff versus client agreement in 10 tobacco-related items

*Client question*	*Smoking clients (N=341) n (%)*	*Staff question*	*Clinical staff (N=183) n (%)*	*p*
1. The hazards of smoking have been clearly demonstrated		1. The hazards of smoking have been clearly demonstrated		
**Strongly agree or agree**	244 (73.5)		146 (82.0)	0.008
2. Counseling for quitting smoking is an important part of this program’s mission		2. Counseling for quitting smoking is an important part of this program’s mission		
**Strongly agree or agree**	138 (41.6)		96 (54.9)	0.001
3. Clients that are smoking in this program want to quit		3. My clients want to quit smoking		
**Strongly agree or agree**	93 (28.3)		63 (35.4)	0.020
4. My clinician or counselor has the required skills to help people in this program quit smoking		4. I have the required skills to help my clients quit smoking		
**Strongly agree or agree**	112 (34.0)		86 (49.4)	0.003
5. Smoking is a personal decision which does not concern my counselor or clinician		5. Smoking is a personal decision which does not concern me as a counselor or clinician		
**Strongly agree or agree**	55 (16.6)		59 (33.1)	<0.001
6. I am concerned about smoking		6. My clients are concerned about smoking		
**Strongly agree or agree**	196 (59.4)		82 (46.6)	0.002
7. Counseling by a clinician at this program would help me to quit		7. Counseling by a clinician helps motivate clients to quit		
**Strongly agree or agree**	115 (34.9)		119 (65.4)	<0.001
8. In your current treatment program, did any staff member ask you whether you smoke?		8. In the past month, how frequently did you ask your clients whether they smoked?		
**Yes, always or very often or often**	219 (64.4)		76 (44.4)	<0.001
9. In your current treatment program, did you receive a referral to a free quitline?		9. In the past month, how frequently did you refer clients to the state quitline?		
**Yes, always or very often or often**	82. (24.2)		36 (22.4)	0.082
10. In the past month, how frequently did your clinician or counselor at your current treatment program encourage you to use products to help you quit smoking (such as nicotine gum, patch, zyban, Chantix)		10. In the past month, how frequently did you encourage clients who smoke to use a nicotine replacement therapy product or other products (such as, zyban, Chantix)		
**Always or very often or often**	79 (23.4)		48 (28.4)	0.040

*Smoking cessation behavior*

Two measures on the client survey were used to assess smoking cessation behavior. Clients were asked if they had voluntarily quit smoking for at least 24 hours at their current program (yes/no) and if they planned to quit smoking in the next 30 days (yes/no). Clients who reported planning to quit in the next 6 months, and those who reported not thinking of quitting at all, were categorized as not planning to quit in the next 30 days.

### Statistical analysis

We describe participant sociodemographic characteristics and tobacco use for all clients, for clients who are current smokers, and for clinical staff using frequencies and percentages (Table 1). Of the 10 tobacco-related knowledge, attitudes, and practices/services items asked of both staff and clients, 8 were directly comparable with the same response codes (Table 2). For item number 8 the response codes were different, but to make these more comparable, we report the proportion of clients responding ‘yes’ with the proportion of staff who responded with ‘always’ or ‘very often’ or ‘often’, combined. To test differences between staff and clients’ responses across the 18 programs we used chi-squared analyses.

We conducted an ecological analysis, with the programs as the unit of analysis, to assess the relationship between staff and client responses on the 10 survey items and voluntarily having a quit attempt during their stay in the treatment program and planning to quit in the following 30 days. The purpose of the Pearson’s correlation analysis was to assess the strength and direction of the association of these variables. Statistical significance was set at p<0.05.

## RESULTS

### Sociodemographic and smoking characteristics

The sample included 535 clients and 183 clinical staff. Across all the programs, 78% of eligible clients and 87% of eligible staff participated, with a participation rate in individual programs ranging from 64% to 100% for clients and 68% to 92% for staff. Table 1 summarizes the sociodemographic characteristics of clients and clinicians. In our sample, 63.7% of clients (n=341) were current cigarette smokers, and 31.0% consumed little filter cigars or cigars, and 33.0% e-cigarettes in combination with manufactured cigarettes. Clients who smoked cigarettes used a mean of 9.9 CPD (SD=7.6). Overall, approximately one-third of cigarette users reported that they were planning to quit smoking in the next 30 days. Regarding clinicians, about 62.3% had consumed a tobacco product in the past month, 21.2% were current cigarette users and 20.8% used e-cigarettes. Cigarette users used a mean of 9.5 CPD (SD=6.5), and 40.5% of cigarette users reported that they were seriously thinking of quitting in the next 30 days.

### Comparison of staff and clients on tobaccorelated survey items

Most clients (73.5%) agreed that the hazards of smoking have been clearly demonstrated, while 82.0% of clinical staff had the same opinion (p=0.008, Table 2). Moreover, while 41.6% of smoking clients agreed that counseling for quitting is an important part of the mission of their program, 54.9% of clinicians did (p=0.001). In addition, 28.3% of smoking clients affirmed that clients want to quit smoking while 35.4% of clinicians affirmed so (p=0.020).

About one-third of clients (34.0%) agreed that my clinician or counselor has the required skills to help smokers to quit compared with 49.4% of clinicians who said they possessed these skills (p=0.003). Few clients (16.6%) considered that smoking is a personal decision and does not concern my counselor or clinician, while 33.1% of clinicians considered client smoking behavior does not concern the counselor (p<0.001). Moreover, 34.9% of smoking clients agreed that counseling by a clinician at this program could help me to quit, whereas 65.4% of clinicians believed that they could help clients quit smoking (p<0.001).

Regarding tobacco-related services provided by staff and reported by clients, most clients (64.4%) reported having been asked whether they smoked in their treatment program, while 44.4% of clinicians said that they always, very often and often ask their clients whether they smoke (p<0.001). Moreover, 23.4% of smoking clients affirmed having been encouraged to use products to help them quit smoking and similarly 28.4% of clinicians reported that they always or very often recommend NRT to their patients (p=0.040).

### Effects of tobacco related knowledge, attitudes, behaviors and practices/services by staff and clients on patients' smoking behaviors

At the program level (N=18 programs), the proportion of clients reporting that smoking was an important part of the program was positively correlated with the proportion of clients who plan to quit in the next 30 days (r=0.661, p=0.004; item 2 in Table 3). In addition, we found a positive correlation between clients who were in programs in which they were referred to a state quitline and the proportion of those who plan to quit in the next 30 days (r=0.661, p=0.004; item 9 in Table 3). Finally, in programs where both clients and staff expressed that NRT or other cessation medications were encouraged to be used during the program stay, more clients were planning a quit attempt in the next 30 days (clients: r=0.645 p=0.004; staff: r=0.524 p=0.025; item 10).

**Table 3:** Correlation between clients’ smoking behavior in the program responses of clinicians/clients about several tobacco-related items

	*Clients (N=341)*		*Clinicians (N=183)*	
	*% Clients made a quit attempt in program*	*% Clients who plan to quit in the next 30 days*	*% Clients made a quit attempt in program*	*% Clients who plan to quit in next 30 days*
	*r*	*r*	*r*	*r*
	*p*	*p*	*p*	*p*
1. The hazards of smoking have been clearly demonstrated	0.038	0.392	0.032	-0.052
	0.884	0.119	0.899	0.838
2. Counseling for quitting smoking is an important part of this program’s mission	0.372	0.661	0.291	0.429
	0.142	**0.004**	0.242	0.076
3. My clients want to quit smoking Clients that smoke in this program want to quit	-0.040	0.407	0.314	0.326
	0.878	0.105	0.204	0.187
4. I have the required skills to help my clients quit smoking My clinician or counselor has the required skills to help people in this program quit smoking	0.345	0.271	0.428	-0.098
	0.175	0.293	0.076	0.699
5. Smoking is a personal decision which does not concern my counselor or clinician	0.173	0.095	-0.254	0.205
	0.507	0.715	0.308	0.415
6. My clients are concerned about smoking I am concerned about smoking	-0.143	0.322	0.440	0.360
	0.584	0.207	0.068	0.142
7. Counseling by a clinician helps motivate clients to quit Counseling by a clinician at this program would help me to quit	0.409	0.196	0.126	0.172
	0.103	0.450	0.619	0.494
8. In the past month, how frequently did you ask clients if they smoke? In your current treatment program, did any staff member ask you whether you smoke?	-0.171	0.448	-0.067	-0.238
	0.498	0.062	0.791	0.340
9. In the past month, how frequently did you refer clients to the state quitline? In your current treatment program, did you receive a referral to a free quitline?	0.0490	0.462	-0.257	0.041
	0.874	**0.053**	0.302	0.870
10. In the past month, how frequently did you encourage clients who smoke to use a nicotine replacement therapy product or any other product? In the past month, how frequently did your clinician or counselor at your current treatment program encourage you to use tobacco cessation products to help you quit smoking?	0.391	0.645	-0.023	0.524
	0.109	**0.004**	0.929	**0.025**

## DISCUSSION

To our knowledge, this is the first study that assesses clients’ and staff’s insights into tobacco control services in SUD residential programs at the same point and the same services. A low level of tobacco-related services was reported by both clients and clinicians. Therefore, opportunities to quit within SUD treatment programs are infrequently promoted by clinicians or received by clients. Nevertheless, client reports of planning quit attempts were positively correlated with the agreement of staff and clients that the use of NRT was encouraged in the programs.

Consistent with prior research, we found high levels of cigarette smoking in residential treatment programs, for both clients (63.7%) and staff (21.2%). These smoking rates may be compared with those in the general population both for the US (19.0%)^28^ and in California (11.2%)^29^ where the study was conducted. In this study, we consider not only the use of combustible cigarettes, but also the use of other tobacco products since although cigarettes are still the most consumed tobacco product, there is an increasing tendency to use other tobacco products alone or in combination^28^. Nevertheless, all clients who reported having used any type of tobacco product in the last 30 days also used cigarettes, while not all the staff who reported using tobacco products also smoked cigarettes.

When we compared staff versus smoking clients’ responses on 10 tobacco-related issues, we observed that clinical staff were more likely to think that quitting smoking is an important part of this program mission versus smoking clients. On one hand, clinical staff were more likely to think that clients want to quit smoking than clients reported to be willing to do so. On the other hand, clients were more inclined to affirm that they were concerned about their smoking than what staff believed clients were. The reasons for these discrepancies were not explored in this work, but results from previous qualitative research have pointed out how, according to smokers in SUD treatment, smoking could be considered a trigger to drug relapse^30^ and suggest finding an optimal time for individual tobacco treatment components, including providing tobacco cessation products (e.g. NRT, bupropion, varenicline) to treat acute withdrawal symptoms^30^.

We observed that <30% of clinical staff reported encouraging patients to use tobacco cessation products. Along the same line, relatively few patients (23.4%) affirmed having been encouraged to use these products. These results, reported by clinicians and validated by patients, indicate that tobacco treatment was infrequent in the clinics studied. Most smoking clients said they were asked about whether they smoke, but they were not confident in their clinician’s skills to help them to quit, or the effectiveness of their clinician’s abilities to motivate them to quit. While half of the staff had confidence in their own ability to help clients quit smoking, clients had less confidence that staff could help them quit. Some research has suggested that clients consider treatment programs an ideal moment to start a quitting process, but were also concerned that doing so may jeopardize recovery^31^. As pointed out by Moore et al.^32^, the competence of acquiring skills such as delivering smoking cessation practices should be evaluated beyond assessing self-reported knowledge. It is also required to assess the level of competence in performing counseling and applying the tobacco cessation protocols in place, to be able to ascertain the level of competencies acquired by each provider^32^. Further research in smoking cessation skill acquisition is needed, since the majority of studies have evaluated self-reported competencies without the use of objective competence measures^33,34^. In addition, clients observe one-third of their providers using tobacco products and almost quarter using cigarettes. This is consistent with a culture of smoking in SUD treatment, and tobacco-free policies may offer one approach to addressing smoking in these settings^20^.

Previous studies have highlighted the low level of tobacco cessation services provided in behavioral health settings in the US^35,36^. Barriers identified by clinicians include limited time, difficulty in engaging smokers, and the perception that clients are not interested^37,38^. Nevertheless, people accessing SUD programs who smoke are motivated to quit but frequently perceive a lack of support from professionals^39^, and this generates missed opportunities to quit^40^. The current study offers an additional point of view on the scarceness of systematic provision of tobacco cessation counseling in SUD programs, related to the contrast between staff and client opinions. Specifically, and compared to staff confidence levels, clients had lower confidence in counselor’s ability to help them quit smoking. This could be a consequence of the low level of smoking cessation support offered by staff and received by clients, as well as the high prevalence of smoking, or other tobacco use, among clinical staff. Strategies to address tobacco use in SUD treatment may include promoting comprehensive tobacco control policies including support for tobacco cessation among clinicians, promoting tobacco-free grounds, and systematically delivering smoking cessation services to smokers, no matter of their level of motivation. In our study sample, it seems clear that there was an imbalance between expectations and provisions of tobacco cessation support, because at least one-third of smokers attempted to quit but a lower percentage received clear support (by being offered NRT or being referred to state quitlines).

Encouraging quitting among smokers in SUD treatment settings, and offering smoking cessation treatment, does not impact negatively on success of abstinence from other substances^4^. Additionally, as clinicians report having the skills to provide smoking cessation but clients lack confidence in their clinicians’ skills, both training and organizational support for tobacco cessation services are needed^41^. The integration of such practices and policies will improve the view of SUD treatment programs as organizations that promote the wellbeing of both workers and clients^36^. At present, very few projects have used implementation strategies to accelerate the uptake of evidence-based smoking practices in SUD treatment programs^41^.

At the ecological level (in which programs were the unit of analysis), we observed associations between client opinion about the importance of tobacco cessation in the program and receiving encouragement to use NRT in the program, to an increase in the number of clients who plan to quit smoking in the next 30 days. This finding highlights the importance of embedding tobacco cessation services in programs and suggests that doing so may have impacts at the individual level. In addition, we observed that programs where staff believed their clients were concerned about smoking, and offered tobacco cessation products to smokers, had more people who planned to quit in the next 30 days. This emphasizes the importance of providing tobacco cessation medication, and that staff should be mindful of how smokers are worried about their smoking.

### Limitations

Study limitations include the small number of programs included (18 residential programs). Nevertheless, we were able to survey more than 180 clinicians and 340 smokers enrolled in these programs. Generalizability may be limited as all participating programs were from California, which has a robust statewide tobacco control program and where the rate of smoking is one of the lowest among US States. All data, from both staff and clients, were self-reported and may be open to recall or other self-report bias. In addition, we were not able to report all the descriptive data about the participants (both clients and staff) such as nicotine dependence and other sociodemographic variables such as marital status or socioeconomic status. However, this study is one of a few comparing the opinions of staff versus smoking clients regarding tobacco-related questions. In addition, we conducted an ecological analysis to assess the effect of these opinions on tobacco consumption behavior among smokers enrolled in these programs.

## CONCLUSIONS

In this study, clients and staff agreed that tobacco cessation services were scarce in SUD treatment program, but their views were discrepant in other areas. While less than one-third of smokers agreed that their providers had the necessary skills to provide smoking cessation, more than half of clinicians self-reported having these competencies. In addition, a lower percentage of smoking clients said that smoking was an important part of the program, while more than half of staff stated that it was. This may influence how smoking clients rely on their clinicians to help them quit. In the programs where clients believed that tobacco cessation was an important part of the program, and who said they were encouraged to use tobacco cessation products, showed a moderate positive association with a higher number of clients planning to quit smoking. State and organizational tobacco control policies should be implemented to improve clinicians’ skills in providing smoking cessation, introduce more systematic approaches to smoking cessation, and promote tobacco control policies embedded in the organization to promote and sustain a tobacco-free culture in SUD treatment programs.

## Data Availability

The data supporting this research are available from the authors on reasonable request.
