# Corrigendum: Comparing client and staff reports on tobacco-related knowledge, attitudes, beliefs and services provided in substance use treatment

**DOI:** 10.18332/tid/166050

**Published:** 2023-05-11

**Authors:** Cristina Martínez, Nadra Lisha, Caravella McCuistian, Elana Straus, Kevin Delucchi, Joseph Guydish

**Affiliations:** 1 Tobacco Control Unit, Cancer Control and Prevention Program, Institut Català d’Oncologia-ICO, Barcelona, Spain; 2 Cancer Control and Prevention Group, Institut d’Investigació Biomèdica de Bellvitge-IDIBELL, Barcelona, Spain; 3 Department of Public Health, Mental Health, and Maternal and Child Health Nursing, School of Medicine and Health Sciences, Universitat de Barcelona, Barcelona, Spain; 4 Institute for Health Policy Studies, University of California San Francisco, San Francisco, United States; 5 Centro de Investigación Biomédica en Red de Enfermedades Respiratorias (CIBERES), Madrid, Spain; 6 Department of Medicine, Division of General Internal Medicine, University of California San Francisco, San Francisco, United States; 7 Center for Tobacco Control Research and Education, University of California San Francisco, San Francisco, United States; 8 Department of Psychiatry and Behavioral Sciences, University of California San Francisco, San Francisco, United States

**Keywords:** substance use, smoking, tobacco policy, tobacco-related

Corrigendum on:

Comparing client and staff reports on tobacco-related knowledge, attitudes, beliefs and services provided in substance use treatment

By Cristina Martínez, Nadra Lisha, Caravella McCuistian, Elana Straus, Kevin Delucchi, Joseph Guydish

Tobacco Induced Diseases, Volume 21, Issue March, Pages 1–11, Publish date: 24 March 2023

DOI: https://doi.org/10.18332/tid/160974

In the originally published version of the article one more affiliation for the author Cristina Martínez should have been provided. The extra affiliation is now placed as affiliation No. 5: Centro de Investigación Biomédica en Red de Enfermedades Respiratorias (CIBERES), Madrid, Spain, and the enumeration of the rest of the affiliations is increased by 1 in the authors list and in the affiliations section (page 1).

Furthermore, the author’s name Elana Strauss is changed into Elana Straus and the author’s name Kevin Deluchi is now changed into Kevin Delucchi.

The affiliation No. 5 has been added online, and the affiliations’ enumeration and order, and the authors’ names have been also corrected online.

